# Enzyme replacement therapy and hematopoietic stem cell transplant: a new paradigm of treatment in Wolman disease

**DOI:** 10.1186/s13023-021-01849-7

**Published:** 2021-05-21

**Authors:** Jane E. Potter, Gemma Petts, Arunabha Ghosh, Fiona J. White, Jane L. Kinsella, Stephen Hughes, Jane Roberts, Adam Hodgkinson, Kathryn Brammeier, Heather Church, Christine Merrigan, Joanne Hughes, Pamela Evans, Helen Campbell, Denise Bonney, William G. Newman, Brian W. Bigger, Alexander Broomfield, Simon A. Jones, Robert F. Wynn

**Affiliations:** 1 Department of Blood and Marrow Transplantation, Royal Manchester Childrens Hospital, Oxford Road, Manchester, UK; 2 Department of Paediatric Histopathology, Royal Manchester Childrens Hospital, Oxford Road, Manchester, UK; 3 Manchester Centre for Genomic Medicine, Manchester University NHS Foundation Trust, Oxford Road, Manchester, UK; 4 Department of Therapy and Dietetics, Manchester University NHS Foundation Trust, Oxford Road, Manchester, UK; 5 Paediatric Allergy and Immunology Department, Royal Manchester Childrens Hospital, Oxford Road, Manchester, UK; 6 National Centre for Inherited Metabolic Disorders, Childrens Health Ireland at Temple Street, Dublin, Ireland; 7 Department of Haematology and Oncology, Childrens Health Ireland at Crumlin, Dublin, Ireland; 8 Evolution and Genomic Science, School of Biological Sciences, University of Manchester, Manchester, UK; 9 Stem Cell and Neurotherapies Laboratory, Division of Cell Matrix Biology and Regenerative Medicine, University of Manchester, Manchester, UK

**Keywords:** Enzyme replacement therapy (ERT), Dietary substrate reduction (DSR), Gene therapy, Hematopoietic stem cell transplant (HCT), Hemophagocytic lymphohistiocytosis (HLH), Lysosomal storage disorders (LSD), Lysosomal acid lipase (LAL), Wolman disease

## Abstract

**Background:**

Wolman disease is a rare, lysosomal storage disorder in which biallelic variants in the *LIPA* gene result in reduced or complete lack of lysosomal acid lipase. The accumulation of the substrates; cholesterol esters and triglycerides, significantly impacts cellular function. Untreated patients die within the first 12months of life. Clinically, patients present severely malnourished, with diarrhoea and hepatosplenomegaly, many have an inflammatory phenotype, including with hemophagocytic lymphohistiocytosis (HLH). Hematopoietic stem cell transplant (HCT) had been historically the only treatment available but has a high procedure-related mortality because of disease progression and disease-associated morbidities. More recently, enzyme replacement therapy (ERT) with dietary substrate reduction (DSR) has significantly improved patient survival. However, ERT is life long, expensive and its utility is limited by anti-drug antibodies (ADA) and the need for central venous access.

**Results:**

We describe five Wolman disease patients diagnosed in infancy that were treated at Royal Manchester Children's Hospital receiving ERT with DSR then HCTmultimodal therapy. In 3/5 an initial response to ERT was attenuated by ADA with associated clinical and laboratory features of deterioration. 1/5 developed anaphylaxis to ERT and the other patient died post HCT with ongoing HLH. All patients received allogeneic HCT. 4/5 patients are alive, and both disease phenotype and laboratory parameters are improved compared to when they were on ERT alone. The gastrointestinal symptoms are particularly improved after HCT, with reduced diarrhoea and vomiting. This allows gradual structured normalisation of diet with improved tolerance of dietary fat. Histologically there are reduced cholesterol clefts, fewer foamy macrophages and an improved villous structure. Disease biomarkers also show improvement with ERT, immunotherapy and HCT. Three patients have mixed chimerism after HCT, indicating a likely engraftment-defect in this condition.

**Conclusion:**

We describe combined ERT, DSR and HCT, multimodal treatment for Wolman disease. ERT and DSR stabilises the sick infant and reduces the formerly described prohibitively high, transplant-associated mortality in this condition. HCT abrogates the problems of ERT, namely attenuating ADA, the need for continuing venous access, and continuing high cost drug treatment. HCT also brings improved efficacy, particularly evident in improved gastrointestinal function and histology. Multimodal therapy should be considered a new paradigm of treatment for Wolman disease patients where there is an attenuated response to ERT, and for all patients where there is a well-matched transplant donor, in order to improve long term gut function, tolerance of a normal diet and quality of life.

## Background

Infantile onset lysosomal acid lipase (LAL) deficiency, also known as Wolman disease, is a rare lysosomal storage disorder (LSD) with an estimated incidence to affect 1:100,000 births [1]. Severe LAL (acid esterase) deficiency leads to the lysosomal accumulation of substrates, cholesteryl esters and triglycerides, which would otherwise have been hydrolysed into free cholesterol and free fatty acids. Untreated, infants are unlikely to survive beyond the first year of life [2].

Wolman disease is caused by the biallelic inheritance of pathogenic variants in the *LIPA* gene on chromosome 10q23.2-23.3 [3]. Case reports have identified multiple loss of function variants that result in a Wolman phenotype, including complete gene deletion. There are some hypomorphic variants that allow some residual LAL activity, and these are often associated with a more attenuated phenotype and later presentation, known as cholesteryl ester storage disorder.

Wolman disease presents in early infancy with intestinal failure and severe malnutrition [2]. Hepatosplenomegaly is common, with progressive liver failure. An incidental radiological finding of calcified adrenal glands is often reported, and is considered pathognomonic for the disease [4]. Vacuolated lymphocytes are visible in the blood smear and patients may also present with pancytopenia and hyper-inflammation, with hypercytokinaemia, and hemophagocytic lymphohistiocytosis (HLH) [5].

**Fig. 1:**
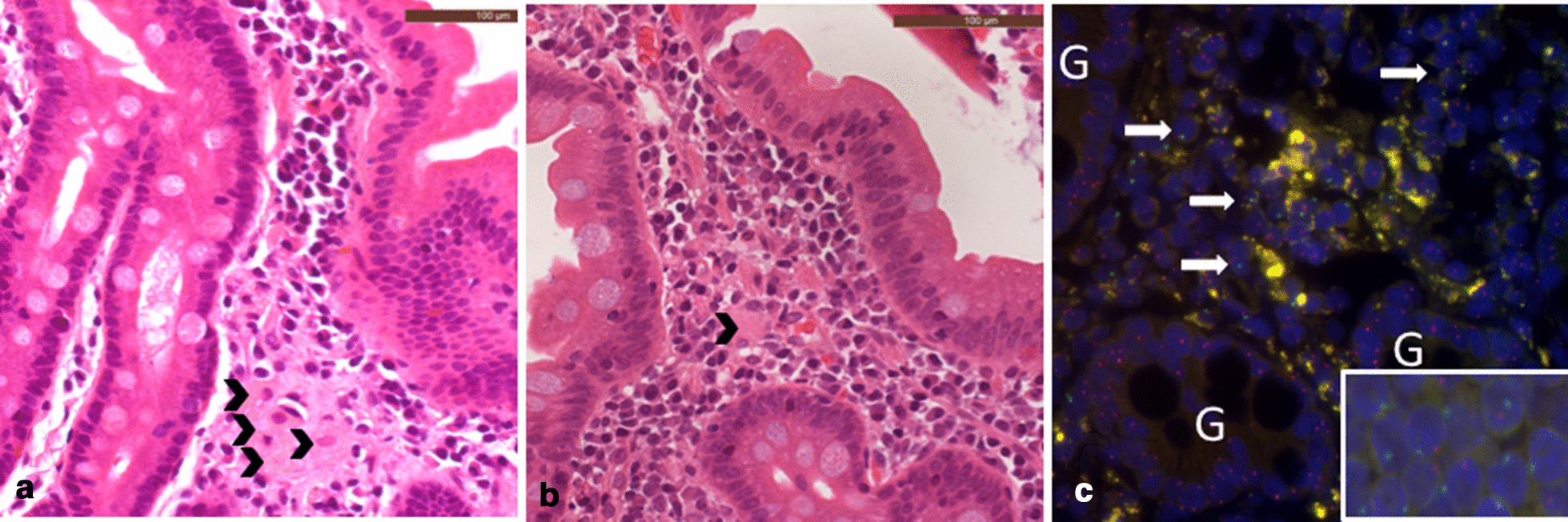
Patient 2 duodenal biopsies post HCT showing improvement overtime. **a** Was taken in 2017, **b** was taken in 2019. **b** shows reduced numbers of foamy macrophages in the lamina propria (arrowheads) and repopulation by lymphoplasmacytic cells (both haematoxylin and eosin stained FFPE sections,400 original magnification). **c** Is duodenal tissue from Patient 2 examined using X/Y chromosome FISH. The X probe is red and the Y probe is green. The patient was female (XX) and the HCT donor was male (XY). The native duodenal glands (G) show the presence of only red X probes but donor cells in the lamina propria show both red X and green Y probes (arrows) (insert is control male tissue showing both probes)

**Fig. 2:**
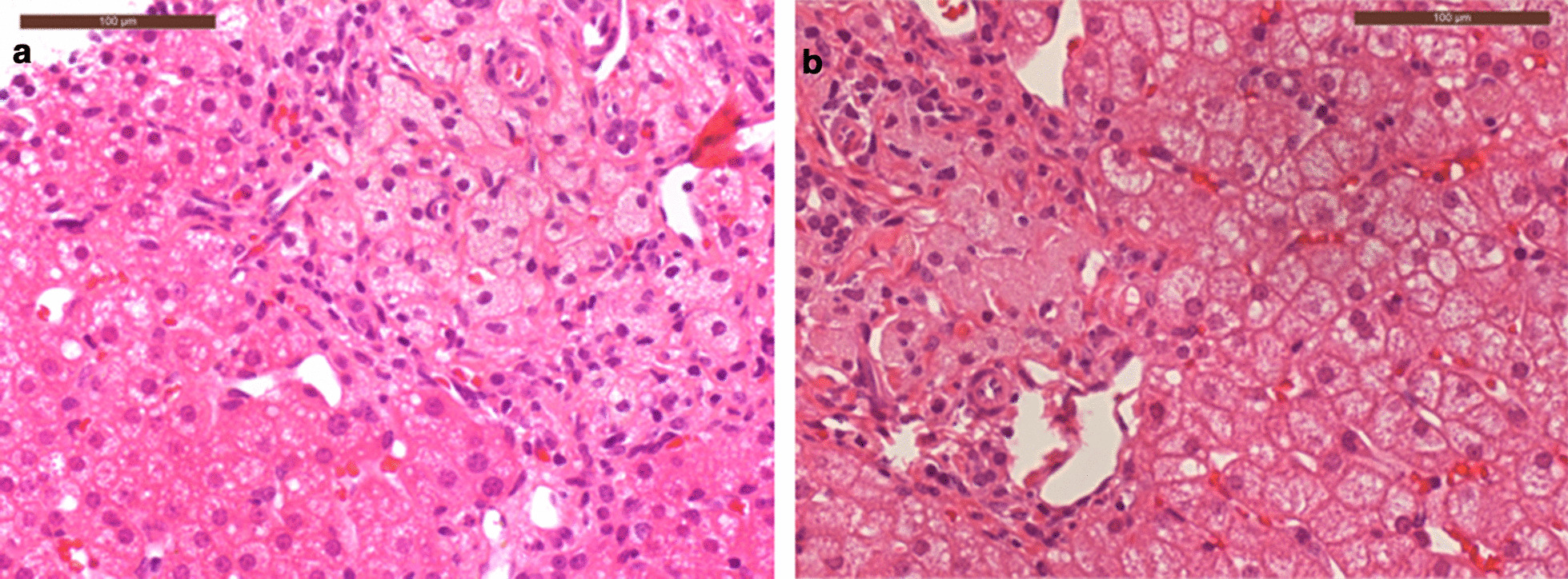
Patient 2 liver biopsies post HCT showing improvement over time. **a** Was taken in 2017, **b** was taken in 2019. **b** shows reduced numbers of portal tract foamy macrophages (both haematoxylin and eosin stained FFPE sections,400 original magnification)

**Fig. 3:**
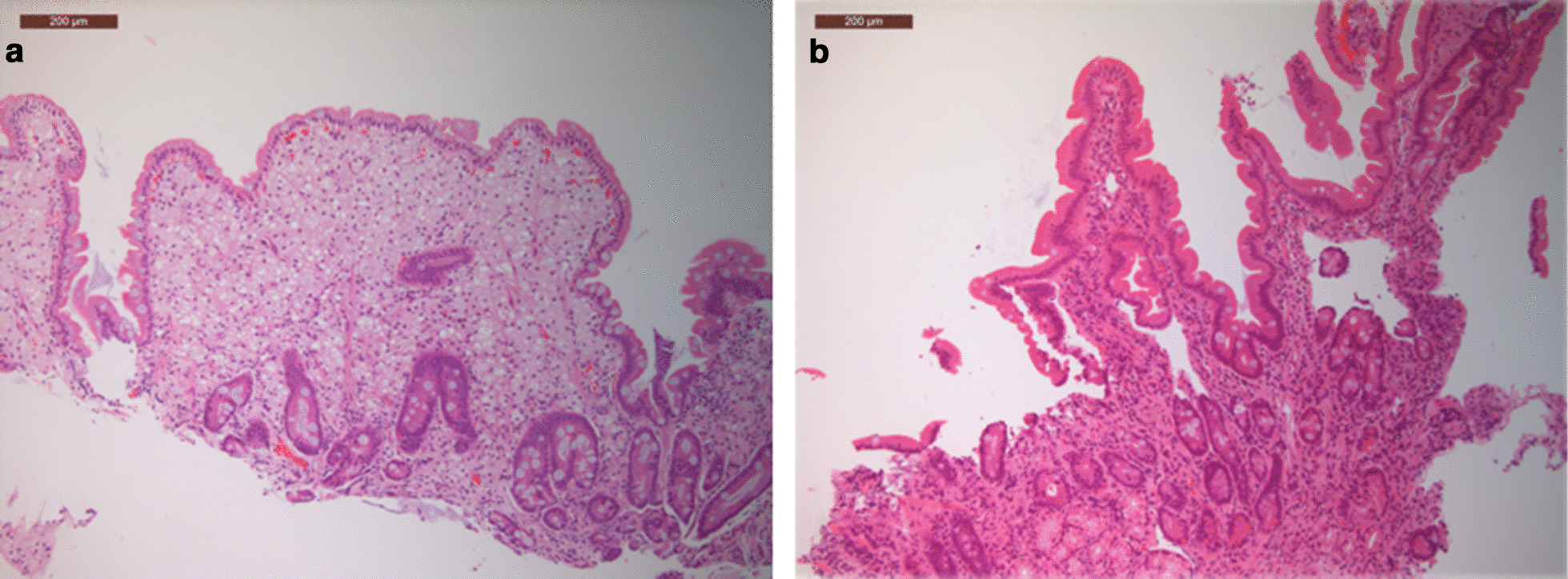
Patient 3 duodenal biopsies pre (**a**) and post (**b**) HCT showing improvement overtime. **a** The pre HCT biopsy shows extensive replacement of the lamina propria by foamy macrophage with broadening of villi. In comparison, the post HCT biopsy **b** shows an essentially normal villous structure with only a few foamy macrophages in the lamina propria (both haematoxylin and eosin stained FFPE sections,100 original magnification)

**Fig. 4:**
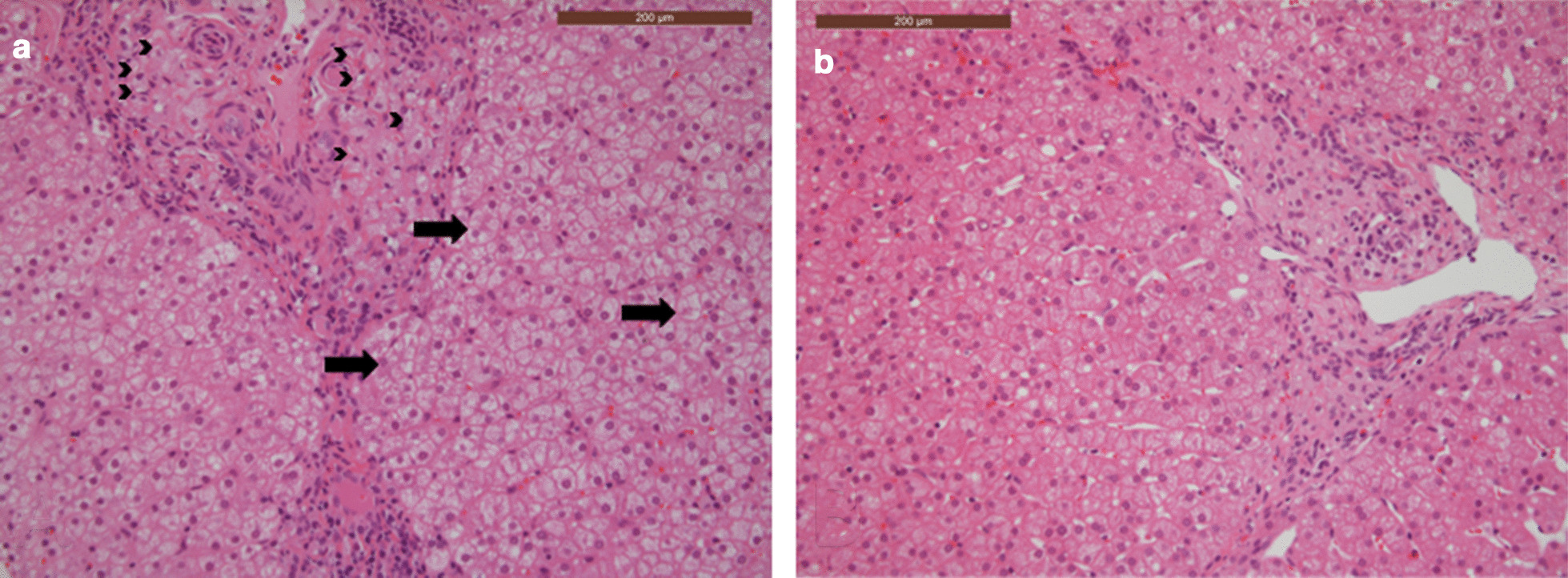
Patient 3 liver biopsies pre (**a**) and post (**b**) HCT showing improvement over time. The post HCT biopsy **b** shows reduced numbers of portal tract foamy macrophages, including those containing cholesterol cleft (arrowheads) and reduced portal inflammation. Early hepatocellular ballooning is seen in the pre HCT biopsy (**a**) but not in the post HCT biopsy (**b**, arrows) (both haematoxylin and eosin stained FFPE sections,400 original magnification)

**Fig. 5:**
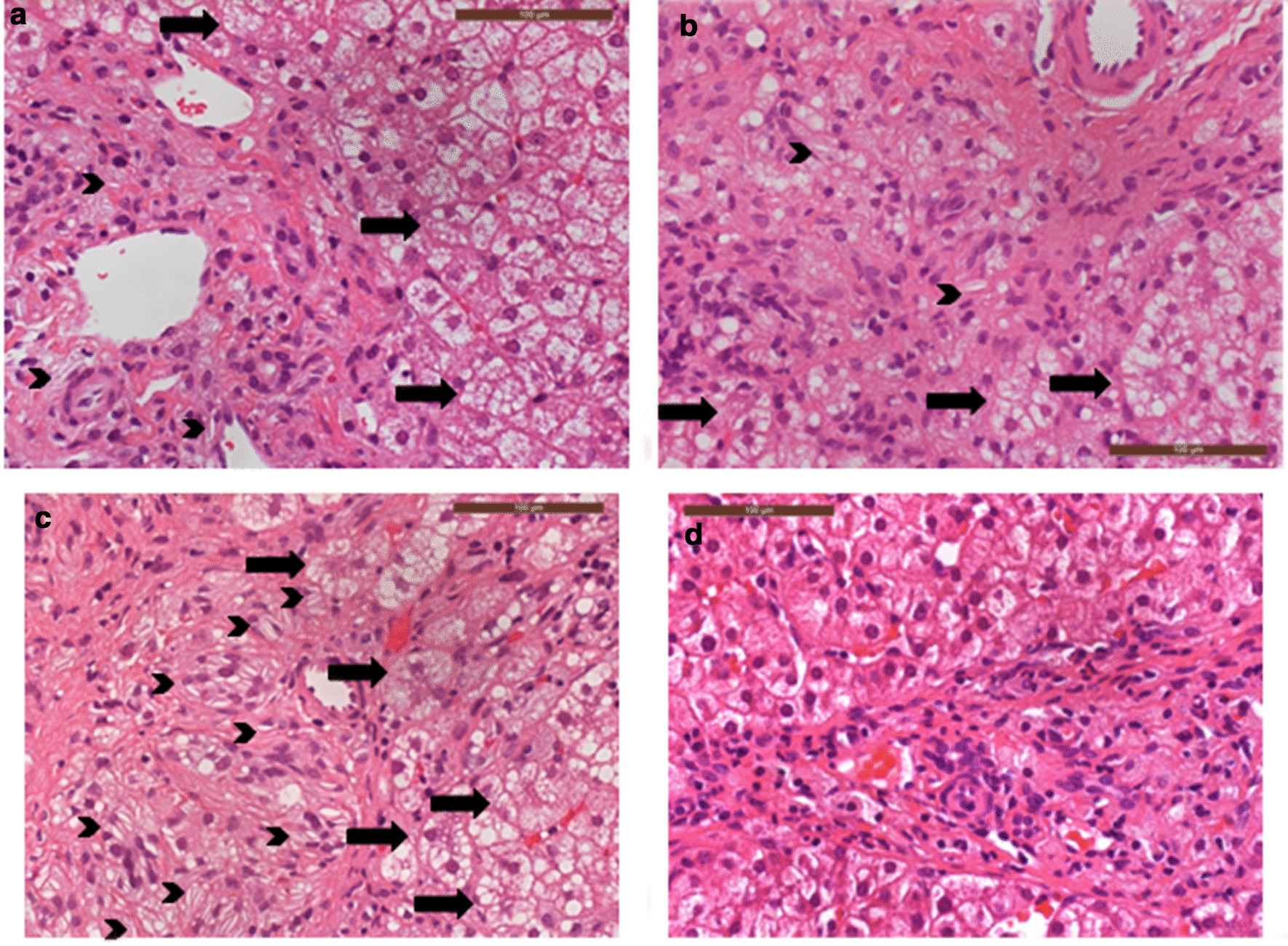
Patient 5 liver biopsies pre and post HCT showing improvement over time. **ac** were pre HCT, taken in 2017, 2018 and 2019 respectively. **d** Was taken in 2020, 12months after HCT. **ac** demonstrate worsening portal tract foamy macrophage accumulation, including many containing abundant cholesterol clefts (arrowheads); there is also worsening hepatocellular ballooning (arrows) and fibrosis. **d** Demonstrates a dramatic improvement in histological appearances after HCT (all images haematoxylin and eosin stained FFPE sections,400 original magnification)

The initiating pathology of all presentations is lysosomal substrate accumulation, including within the resident macrophages of the gut and liver, which can readily be observed in tissue biopsies (Figs.1, 2, 3, 4, 5). The inflammatory sequelae and their relationship with substrate accumulation remain incompletely understood, but likely involves macrophage inflammasome activation by accumulated cholesteryl esters [5]. A biochemical diagnosis is achieved by demonstrating deficiency of LAL activity, usually within the leukocytes. High levels of specific oxysterols (including cholestane-3,5,6-triol), which are intermediates of cholesterol metabolism produced under oxidative stress, have been found to be associated with initial presentation and clinical deterioration [6, 7].

Until recently, treatment options have been very limited, and Wolman disease was fatal in infancy [2, 8]. Dietary substrate reduction (DSR) is achieved by minimal or fat free diet, but does not control substrate accumulation sufficiently as a treatment on its own, due to ongoing endogenous synthesis. Current treatment options include allogeneic hematopoietic stem cell transplant (HCT) and enzyme replacement therapy (ERT) combined with DSR. In this report we discuss the use of combining both approaches, a multimodality therapy for Wolman disease, to achieve optimal patient outcomes.

### Treatments

The deficient enzyme can be delivered to enzyme-deficient cells in a process known as cross correction. Exogenous enzyme, (derived from donor leukocytes in HCT or prepared as a pharmacological drug product in ERT) is taken up by receptor-mediated endocytosis and is directed to the lysosome through the association of mannose and mannose-6-phosphate residues on the enzyme, and corresponding receptors on the cell surface. In this way, exogenous enzyme can be specifically trafficked to the substrate-laden lysosome. Uniquely in Wolman disease, DSR can be manipulated allowing for a third, complementary modality of treatment.

### Hematopoietic stem cell transplant (HCT)

Engraftment of donor leukocytes following HCT requires conditioning chemotherapy to both ablate recipient marrow to create space for donor marrow, and to suppress the host immune system to prevent rejection of donor cells. Untreated Wolman disease patients are poor candidates for HCT as they are severely malnourished, frequently present with fever, with an ongoing inflammatory process and have significant, rapidly progressive liver disease. HCT in Wolman disease has a prohibitively high treatment-related mortality [2, 9,11]. There are some reported cases of successful outcomes, indicating the potential utility of an HCT approach.

In 2009, four Wolman disease patients reported by a single centre underwent HCT of which two died, and two survived HCT [12]. The elder surviving patient was 11years old at the time of the report and was clinically well, with mild to moderate neurocognitive impairment, attributed to the treatment course rather than the disease itself. The other surviving patient was 4years old, and had normal neurocognitive development. Two patients died of transplant-related complications, 67days and 8months respectively, after transplant, despite engraftment of donor cells [12]. In 2016, an extensive, multi-centre, retrospective international cohort study of 35 patients with Wolman disease included 10 patients who had received HCT, one of whom had also undergone a liver transplant. All 35 patients died, with a median survival age of 3.7months untreated, and 8.6months in the HCT group [2].

### Enzyme replacement therapy (ERT)

Sebelipase alfa (Kanuma), is a recombinant form of LAL (Alexion Pharmaceuticals, Inc., New Haven, Connecticut, USA). A recent study that published data from the VITAL study (n=9) and CL-08 study (n=10) combined survival data of Wolman disease patients on ERT and DSR. 79% of patients survived to aged 12months, with a 68% likelihood of surviving to 5years of age. However, this includes patients who had also received HCT, and not just those on ERT and DSR alone. The combined data also assesses functional development of the children through the trial which remained stable throughout [13].

Initial data indicates that although ERT and DSR have utility in controlling disease progression, they are limited by the development of anti-drug antibodies, the high cost and the need for central venous access for life-long weekly enzyme infusions. ERT is not curative, and residual disease manifestations remain. For example, most of the surviving patients reported in the combined studies continued to require highly modified specialised diets [13].

As seen with ERTs for other LSDs, immunogenicity in the form of ADAs is a concern [14]. In total 10/19 patients in the VITAL and CL-08 study tested positive for ADAs.In CL-08, 6/10 ADAs persisted, and were considered to be neutralising, affecting enzyme uptake and activity. In 3/6, ADAs were considered clinically significant and were associated with reduced ERT efficacy, such that HCT and/or immunomodulatory therapy was given. Of the cases discussed in this review, 3/5 patients had significant ADAs as part of their indication for HCT [13].

We report the outcomes of a group of patients who received multimodality therapy, including ERT, DSR and HCT.

## Results

### Multimodality therapy in Wolman disease patients at Royal Manchester Childrens Hospital (RMCH)

Royal Manchester Childrens Hospital (RMCH) is a specialist centre for diagnosis and treatment of inherited metabolic disorders including delivery of HCT. Since 2005, 15 patients with Wolman disease have been treated in Manchester. Of these, 8 individuals have died: 1 received neither ERT or HCT; 1 patient received HCT but died from disease progression before engraftment of donor cells and 3 patients died of disease having only just commenced ERT (receiving 18 doses). Two patients died on long term ERT, 1 from central line complications and 1 developed ADAs and died aged 3years and 9months with progressive portal hypertension and cirrhosis of the liver. Of the 8 patients that died 1 received multi modal therapy. Seven patients are currently alive; of these all received ERT and DSR; 3 remain on ERT and DSR alone, and 4 have required additional HCT. The 5 patients in the multi modal group (ERT, DSR and then HCT) that will be discussed in more detail. A summary of this group of patients can be seen in Table 1. None of the patients in the multimodal group are related to each other.

**Table 1:** Summary of RMCH multimodal group (patient 15)

	Patient 1	Patient 2	Patient 3	Patient 4	Patient 5
Age at start of ERT (months)	4	2	1	4	3
Age at HCT (months)	8	25	31	7	48
LAL activity at diagnosis (nmol/mg/h)	51	65	51	93	44
Pathogenic variant	Missense variant. homozygous	Deletion: chr10:90,947,44991,060,391 (112kb) (hg19). Complete *LIPA* gene deletion. homozygous	Deletion: chr10:90,947,44991,060,391 (112kb) (hg19). Complete *LIPA* gene deletion. homozygous	c.684delT [p.(Phe22Leufs*13)] *LIPA* exon 4 deletion (frameshift)compound heterozygous	Deletion: chr10:90,947,44991,060,391 (112kb) (hg19). Complete *LIPA* gene deletion. homozygous
Indications for HCT	Suboptimal response to treatment and ongoing HLH	Suboptimal response to treatment with ADA and poor central venous access	Suboptimal response to treatment with ADA	Intolerance to ERTanaphylaxis	Suboptimal response to treatment with ADA
Conditioning	Treosulfan, cyclophosphamide, ATG	Treosulfan, Thiotepa, Fludarabine, Alemtuzumab	Treosulfan, Thiotepa, Fludarabine, Alemtuzumab	Treosulfan, Thiotepa, Fludarabine, Alemtuzumab	Treosulfan, Thiotepa, Fludarabine, ATG
Donor cell source	PBSC matched family	Bone marrow matched sibling	PBSC haploid and CD19 deplete	Bone marrow matched unrelated	Umbilical cord matched unrelated
HCT acute complications	Mild VOD	Nil	Nil	Mild VOD, acute skin GvHD (grade 2)	Engraftment syndrome, acute skin GvHD (grade 2)
Survival (age as of August 2020)	Died aged 13months	Alive6years 5months old	Alive5years 3months old	Alive2years 8months old	Alive4years 10months old
Peripheral blood chimerism 4w post HCT	100%	100%	100%	100%	100%
Peripheral blood chimerism current	NA	26.3% static	30.2% static	14.5% static	100%
Current LAL activity (nmol/mg/h)	NA	200	165	101	469
Current ERT dose post HCT	NA	1mg/kg alternate weeks	3mg/kg alternate weeks	5mg/kg weekly	3mg/kg weekly

*ADA* antidrug antibody, *ATG* anti thymoctye globulin, *ERT* enzyme replacement therapy, *GvHD* graft versus host disease, *HCT* hematopoietic stem cell transplant, *HLH* hemophagocytic lymphohistiocytosis, *LAL* lysosomal acid lipase, *PBSC* peripheral blood stem cells, *VOD* veno occlusive disease. Normal reference range for leukocytes LAL enzyme is 3502000nmol/mg protein/h

**Fig. 6:**
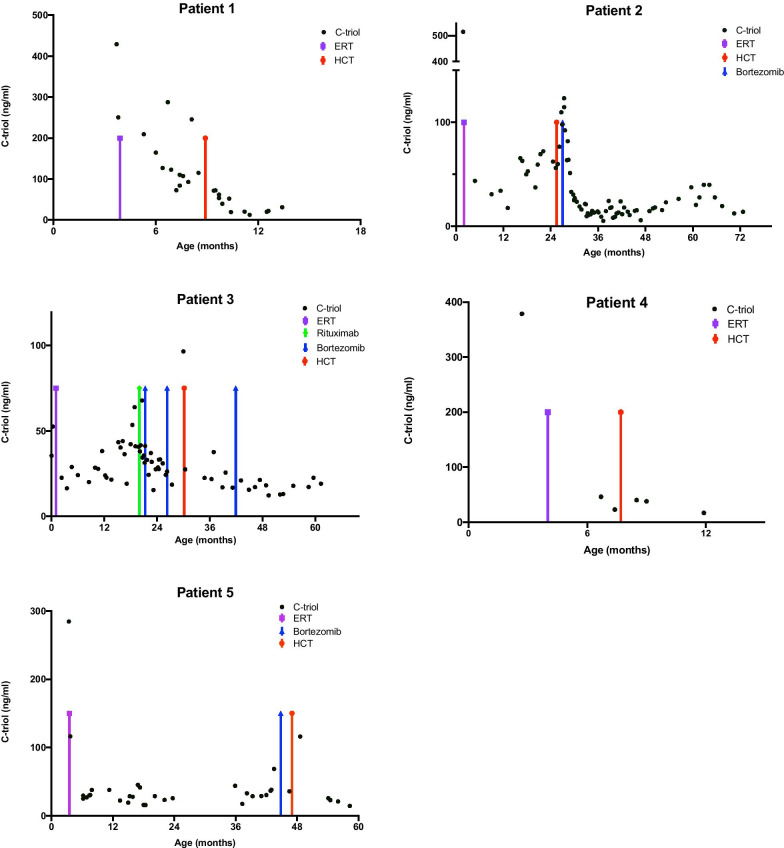
OxysterolCholestane-3,5,6-triol (C-triol) levels overtime per patient 15. Demonstrates improvement in inflammation/C-triols after HCT. The purple arrow highlights the start of ERT, the red arrow highlights the timing of HCT, blue arrows the use of bortezomib and the green arrows rituximab

At the point of HCT, these 5 patients were older than they would have been for HCT had ERT not been available, and clinically their nutritional status and liver function were better than at presentation, reducing the procedure-related mortality associated with HCT. Although the ERT efficacy had reduced, it had facilitated their bridging to HCT and likely improved their HCT survival. Those with high titre ADAs associated with clinical deterioration received increasing dose of ERT and immunotherapy such as bortezomib, a proteasome inhibitor, to improve efficacy of ERT. The initial positive effects of the immunotherapy became less effective demonstrated with worsening histology and increases in oxysterols (Fig.6). Four of these patients are alive and remain engrafted with donor-derived leukocytes in the peripheral circulation.

### Key result outcomes

**Fig. 7:**
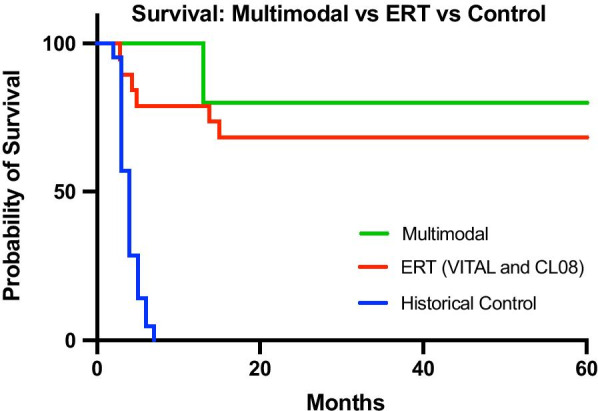
Kaplan Meier survival of the multimodal group versus ERT [13] versus untreated historical Wolman disease control [2, 15]. Survival is estimated from birth to 60months. Historical control data and ERT data has been used with permission from the lead authors of the respective publications (Dr. Simon Jones and Dr. Suresh Vijay respectively)

1. 4/5 patients who received HCT after ERT and DSR are alive. Their survival compares well to those on ERT (Fig.7).
2. All surviving patients have improvement in gastrointestinal symptoms not seen with ERT and DSR. This is evident both clinically in improvements in their dietary tolerance and resolution of diarrhoea (Table 2), and histologically in their biopsies (Figs.1, 2, 3, 4, 5).
3. The 4 surviving patients remain on ERT, 3/5 are on less frequent and much smaller doses than pre HCT with no obvious evidence of neutralising ADAs (Table 1).
4. 3/4 surviving patients have mixed chimerism, 5/5 initially had 100% chimerism, but this fell and then plateaued within the first year post HCT in all but one patient (Table 1).

**Table 2:** Summary of dietary management, growth and gastrointestinal (GI) function of the four surviving multimodal patients (patients 25)

Patient	Time since HCT (August 2020)	Growth Z scores for age				Nutritional intake					Time (months) to resolution of gastrointestinal symptoms
		Weight		Height	MUAC	Protein type		Fat (long chain) g/kg/day	Fat (long chain) Total g/day	Feeding route	
2	4years 6months	Pre HCT	0.1	N/A	0.81	Pre HCT	Amino acids	0.2	2.6	Gastrostomy	15
		Post HCT	0.55	0.59	0.79	Post HCT	Intact	1.3	30	Oral and reducing overnight feed (40%)	
3	2years 9months	Pre HCT	0.97	0.33	0.01	Pre HCT	Amino acids	<0.1	0.8	Gastrostomy	7
		Post HCT	0.54	0.07	1.00	Post HCT	Intact	1.4	30	Oral and reducing overnight feed (15%)	
4	2years 1month	Pre HCT	0.94	0.86	0.23	Pre HCT	Amino acids	<0.1	0.8	Oral	1
		Post HCT	1.95	0.70	N/A	Post HCT	Intact	1.7	30	Oral	
5	10months	Pre HCT	0.64	1.00	0.00	Pre HCT	Amino acids	<0.1	0.8	Gastrostomy	10
		Post HCT	0.59	1.40	0.07	Post HCT	Amino acids	0.3	5	Oral/gastrostomy	

*GI* gastrointestinal, *MUAC* mid upper arm circumference

Pre HCT all had had severe GI involvement initially requiring modified, fat restricted total parenteral nutrition (TPN) and were then transitioned to amino acid based minimal fat enteral feeds. In patients 2,3 and 5 there was significant ongoing GI involvement with vomiting and significant diarrhoea. Despite ongoing GI disturbance normal growth was still achievable with weight, length/height and mid upper arm circumference (MUAC) for age Z scores all being within 1 to+1 pre HCT

Immediately post HCT all cases had worsening of vomiting and diarrhoea and all required modified fat restricted TPN and then slow reintroduction of their pre HCT feeds. Vomiting was quicker to resolve than diarrhoea (3 episodes per day) which took up to around 10months to resolve. Resolution of symptoms was quicker in patient 4, who had less GI pre HCT. Once diarrhoea resolved patients were then systematically transitioned from amino acid-based feeds to intact protein e.g. skimmed milk over a period of 48weeks. During this period there was considerable improvement in oral aversion in patients 2 and 3 with them beginning to eat and over time requiring significantly reduced supplementary gastrostomy feeds

Prior to HCT all patients were on minimal fat intakes. Post HCT patients 2, 3 and 4 have had a gradual increase in fat intake and are now eating a normal diet. There has been no change in GI function with increasing dietary fat intake. Growth parameters post HCT in these patients continue, so far, to be normal

All patients with Wolman disease at RMCH are under the care of a multi-disciplinary team including a specialist dietitian. At diagnosis, and pre-HCT, substrate reduction via minimal lipid intake is initiated alongside ERT. Initially as modified total parenteral nutrition, transitioning over several months to amino acid based minimal fat enteral feeds, usually as continuous tube feeds. The majority of patients on ERT alone have remained with a significant fat restriction in their diet reflecting ongoing gut disease and an intolerance to lipid loads. Post HCT, patients 25 all have noticeable improvement in their ability to tolerate normalisation of enteral feeds. Diarrhoea cessation usually occurs within 812months post HCT, once achieved intact protein and the dietary fats can be increased. Over time oral intake has improved (patients 24) and the reliance of tube feeds decreased.

### Patient 1

This patient was diagnosed aged 4months having presented with growth failure, diarrhoea, hepatosplenomegaly and signs of HLH. This patient was commenced on ERT and DSR, liver function and oxysterols improved, but HLH persisted and HCT was carried out 4months after diagnosis. There was evidence of an ongoing inflammatory process post HCT, with persistent fever, cytopenia, splenomegaly and hyperferritinemia, despite engraftment of donor-derived leukocytes. The patient developed sepsis in the context of cytopenia and ongoing immune suppression, and died 5months post-HCT.

### Patient 2

This patient was diagnosed aged 2months and received ERT and DSR shortly after diagnosis. Initially, their nutritional status and liver function improved. However, central venous access became increasingly difficult with multiple line infections. The efficacy of ERT declined over time, with worsening liver function and growth parameters associated with the development of ADAs. HCT was performed at age 25months for both indications. Other than line infections there were few complications during HCT. White cell engraftment was achieved at day 19 with 100% donor chimerism initially, but within the first 12months mixed chimerism developed. Donor levels have now plateaued at around 26%. Post-HCT, the patient remained on ERT, but the dose was decreased and the dose interval was increased (Table 1). At 4years post-HCT, transaminases are mildly raised and serum LDL cholesterol is stable. Growth has been good, the patient now eats predominantly normal food including fats, with minimal diarrhoea or vomiting (Table 2), this improvement is supported by biopsy findings (Figs.1, 2). Although all biopsies were taken post-HCT, both duodenal and liver histology show improvement with time since HCT. It is possible that the gradual improvement seen is related to the replacement of resident macrophages in the duodenum and the liver (Kupffer cells) over time (Fig.1c). The patient attends mainstream school, but recent neuropsychological assessment does show mild intellectual disability of uncertain aetiology. There is a family history of learning difficulty unrelated to Wolman disease.

### Patient 3

The patient was diagnosed aged 1week, due to a known family history of Wolman disease. ERT and DSR was initially effective, but subsequently a deterioration in liver function and worsening feed intolerance, requiring a period of lipid free parenteral nutrition was observed, again associated with an increase in serum ADA. Immunomodulation was given (rituximab and bortezomib) with the aim of mitigating the effects of the ADAs to maximise the efficacy of ongoing ERT pre HCT and thus improving the patients clinical condition to better tolerate HCT. This initially led to a degree of clinical and biochemical improvement, but with repeat courses, ERT efficacy was reduced (Fig.6). HCT was performed aged 31months and the acute HCT course had minimal complications, with the exception of the development of nephrotic syndrome, caused by immune complexes related to ADA and ERT, after treatment with steroid and bortezomib this appears to have resolved. Initial chimerism was 100% but has fallen and is now stable at 30% donor.

Pre and post-HCT, this patient had liver and gut biopsies to monitor disease (Figs.3, 4). Post-HCT this patient had a significant clinical improvement in gut symptoms, which was not seen with ERT alone (Table 2). This is reflected in the histology, where pre-HCT, the duodenal villi are broadened and abnormal, and foamy macrophages and cholesterol clefts can be seen, post HCT the villus structure looks more normal, there has been a reduction in foamy macrophages, and cholesterol clefts are no longer seen (Fig.3). This patient is in main stream school and is at peer level academically. Although reduced in dose and frequency, this patient remains on ERT.

### Patient 4

This patient presented aged 12weeks with fever, pancytopenia and hepatosplenomegaly with associated transaminitis. A diagnosis of HLH was made and the patient was initially treated on the HLH 2004 protocol. ERT and DSR treatment was commenced with associated improvement in clinical condition. On the fifth infusion of ERT, the patient had an anaphylactic reaction. Continuation of ERT was facilitated by the use of omalizumab, an anti-immunoglobulin E antibody, together with slower ERT infusion rates. This regimen was continued until the patient was fit enough for HCT. The HCT acute course was fairly uneventful, with mild veno-occlusive disease, and grade 2 acute GvHD, which were treated with furosemide and prednisolone respectively. As with the previous 2 patients, initial chimerism was 100% but has fallen and is currently static at 14.5%. This patient remains well on weekly ERT, which is well tolerated, with no anaphylaxis.

### Patient 5

This patient presented aged 3months with diarrhoea, growth failure and hepatomegaly with marked transaminitis. After a diagnosis of Wolman disease was established, they were commenced on ERT and DSR, with good effect initially. They developed worsening hepatic fibrosis while on ERT, which was monitored with serial imaging and biopsy (Fig.5). They also developed clinically relevant high titre ADAs (where clinically relevant high titre ADAs are defined as; sustained high titre antibodies with significant uptake inhibition, rising biomarkers and significant deterioration in clinical condition despite maximal ERT dosage and diet fat restriction). Similar to patient 3, a course of bortezomib was given pre-HCT, which improved the patients clinical condition and is reflected in the drop in oxysterols (Fig.6). After initially improving following one course of bortezomib, there was some deterioration and the decision was made to treat with HCT. During HCT, the patient developed a prolonged fever, with persistently negative bacteriology and virology. This was treated as engraftment syndrome with a course of methylprednisolone. There were no positive indicators of HLH. This patient continues to have 100% donor chimerism. The inflammatory response was more prolonged than would be expected in HCT, and indeed the patient continues to have episodes of fever of unknown origin with associated transaminitis. These episodes respond to steroids, although the required dose of steroids has greatly reduced and the frequency of the febrile illnesses has decreased overtime since HCT. Clinically the patients GI symptoms continue to improve and the most recent histology demonstrates huge improvement in the duodenum and the liver (Fig.5).

### Overall survival results

Survival data from our multimodal treatment group are shown alongside, a historical control of untreated Wolman disease patients [2, 15] and survival data from the VITAL and CL-08 studies in Fig.7. A key consideration is the likely overlap between arms as some of the patients within the group that survived on ERT and DSR, also received HCT [13]. The multimodality survival curve demonstrates 80% survival in a difficult group of Wolman disease patients where ERT efficacy was severely reduced.

## Discussion

We describe a series of children who received HCT for Wolman disease, after pre-treatment with ERT and DSR. In other LSDs such as mucopolysaccharidosis I, we have pioneered the combination therapy of ERT and HCT, where ERT given pre- and peri-HCT improves the somatic performance of HCT recipients, making the transplant more tolerable, and without sensitising the recipients against the donor [16]. In the long term, the engrafted donor leukocytes provide a superior enzyme dose to residual enzyme-deficient cells than ERT, and ERT can therefore can hopefully be stopped [16].

We acknowledge limitations to this study; the sample size is small, making statistical analysis challenging. Given the rarity of Wolman disease, little is known about an optimal HCT approach e.g. optimal donor source and conditioning regimen, and more experience is needed here. The retrospective, non-controlled nature of the study has resulted in multiple variables between patients e.g. timing and frequency of tests, and documented clinical events. Comparison in survival was only achievable using available published data.

ERT and DSR in Wolman disease improves the patients clinical status before HCT, making HCT more tolerable and reducing HCT-related mortality [12]. Long term, HCT provides a more cost-effective approach, is not associated with ADA and appears to demonstrate better correction of disease than ERT alone. Histologically and clinically, this is most notable in the gastrointestinal biopsies and symptoms. It is likely HCT provides more enzyme to tissues than ERT, through better distribution, larger/consistent quantities of available enzyme, or both. We further demonstrate that using ERT and DSR as a bridge to transplant appears to improve overall survival.

The multidisciplinary team decision to progress these patients from long term ERT to HCT was due to disease progression while on ERT. ADAs with associated deterioration in clinical status was the most common indication. The patients with significant ADAs in our series all have a complete deletion of the *LIPA* gene, likely predisposing them to an increased susceptibility of developing ADAs. This is similar to the increased risk of ADA development that is well described in patients with cross reactive immunological material (CRIM) negative Pompe patients during ERT [14]. In Wolman disease patients post HCT, ADAs have either not persisted or are at lower levels. There was no evidence of significant uptake inhibition, suggesting that any persistent ADAs post HCT do not have functional impact on the efficacy of donor enzyme.

Although all 5 patients had 100% donor chimerism and were initially fully engrafted, all but one of the surviving patients now has mixed chimerism, only patient 5 continues to maintain full donor chimerism. Mixed chimerism results in only a proportion of the leukocyte population having the ability to express the LAL protein, and thus cross correct surrounding tissue. The reason for the fall in chimerism is not fully understood. Myelopoiesis in LAL-/- mice has not only shown defects in the myeloid progenitor cells, which HCT can potentially correct, but also a suggestion that the microenvironment did not allow normal hematopoiesis [17]. This could explain the decline in chimerism, which does appear to stabilise in each patient overtime. But it is more likely reconstitution of autologous, residual recipient hematopoietic stem cells that "survived myeloablative chemotherapy. Patient 5 is the only patient who has 100% donor chimerism and is the only patient who has cord blood as their donor source. In other LSD we have reported better chimerism from cord blood donors than other cell sources [18]. The 4 surviving patients remain on ERT, albeit less frequent and smaller doses in most cases. It is clear from the histological studies that enzyme delivery is superior following HCT than with ERT alone, and we hope to be able to stop ERT in the transplanted children.

The mechanism of the inflammatory disease in Wolman disease is incompletely understood, as is therefore, the utility of HCT to reverse such disease. Indeed, the only death in this series was 5months after HCT, where sustained donor-derived engraftment was apparently insufficient to control such disease. There are many case reports of HLH and Wolman disease [5, 19] and some suggestion that the cholesteryl ester crystals cause inflammasome activation [5]. Certainly, more research is needed to understand fully the pathophysiology of this Wolman disease associated HLH.

Ex vivo hematopoietic stem cell gene therapy (HSC-GT) has been used in other lysosomal storage disorders including Metachromatic Leukodystrophy, Mucopolysaccharidoses I and Mucopolysaccharidosis IIIA [20,22]. Although in the early stages of clinical use*,* HSC-GT appears safer than HCT, since autologous cells transduced with a lentiviral vector overexpressing the missing transgene are used. This decreases the risk of infection as the need for immune suppression is reduced. There is also a reduction in the risk of Graft versus Host Disease (GVHD) since there is no allogeneic donor. Infection and GVHD constitute the principle risks of allogeneic HCT [21]. HSC-GT is likely to be more effective than allogeneic HCT, since the transgene is inserted under the control of a constitutive or myeloid cell-specific promoter, and supraphysiological enzyme levels are obtained in engrafted leukocytes driving an improved disease response. Since predicted disease response is related to the enzyme output from engrafted cells, it might be that ex vivo HSC-GT would treat Wolman disease even more effectively than the multimodal treatment we describe here.

## Conclusions

Multimodal management of Wolman disease including ERT and DSR as a bridge to HCT aids survival in Wolman disease. ERT and DSR improves the HCT process, and HCT likely provides better enzyme delivery to tissues and better long-term disease outcomes than those achievable with ERT alone. Multimodal treatment (ERT and DSR followed by HCT) should be considered a new paradigm of treatment for Wolman disease patients where there is an attenuated response to ERT, and for all patients where there is a well-matched donor for HCT. This will improve long term gut function, tolerance of a more normal diet and ultimately quality of life.

## Methods

This study was designed to retrospectively analyse multimodal management of Wolman Disease. The primary objective was to demonstrate efficacy of multimodal management when compared to ERT alone and a historical cohort.

### Patients

Patients in the multimodal group were all treated at RMCH and had confirmation of diagnosis of Wolman disease by demonstrating a deficiency of LAL activity in leukocytes and pathogenic variants in the *LIPA* gene respectively. Included patients received both sebelipase alfa and HCT. Data was gathered retrospectively from patient notes and electronic records. 3 of the 5 patients had the same genotype, which included a whole deletion of the *LIPA* gene, to the best of our knowledge none of the patients were related. Of the patients included in the multi modal group, 4/5 were participants in clinical trials of sebelipase alfa.

### Treatments

Multimodal treatment is defined as ERT (sebelipase alfa) with DSR and then HCT. ERT dose was based upon clinical need. During the pre-HCT period doses ranged from 0.35mg/kg once weekly to 7.5mg/kg once weekly, delivered as an intravenous infusion, usually via a central venous catheter. Post-HCT, the frequency and dose of ERT was reduced. HCT, including donor source and conditioning treatment, was allocated as the best option available for the patient at the time of HCT. Conditioning involved immunosuppression and myeloablation, and was modified according to the patient's individual clinical condition and the donor source. All patients received defibrotide as prophylaxis for veno-occlusive disease.

### Anti drug antibodies (ADAs)

ADAs appear to develop rapidly, usually in the first 3months after commencing ERT, and with varied doses of ERT (including as low as 1mg/kg). This observation is consistent with early data concerning ADAs from the ERT clinical trials. More recently functional antibody data has been gathered using an assay developed at Manchester Centre for Genomic Medicine [23].

### Histology

Histology samples within this report were taken according to clinical need, and were not taken according to a structured clinical research regime, as such, the number of samples per patient and timing relative to start of treatments vary. The samples presented are intended to support the clinical report of symptoms, and highlight any subtle difference between ERT and the multimodal group. The histological reports were made with no prior knowledge of intention of retrospective analysis. All images were of formalin fixed paraffin embedded tissue cut at 4 microns and stained with haematoxylin and eosin. Images were obtained using Leica DM4B light microscope, a Leica DFC450 C camera and Leica Application Suite imaging platform.

FISH34m thick paraffin sections of formalin fixed, paraffin embedded diagnostic archival specimens were deparaffinised, pre-treated with HCl and spotlight enzyme solution. Probes (Zytovision ZytoLight CEN X/Yq12 Dual Color Probe) were used as per the manufacturers instructions (co-denaturation on a metal heating block in 72C oven for 5min, hybridisation in a wet box for 1648h in 37C oven). Sections were counterstained with Vectashield Mounting Media with DAPI and coverslipped. Images were captured using Leica DM2500 LED fluorescent microscope, Leica DFC365 FX camera,63 objective and Leica LAS X imaging software.

### Laboratory investigations (Manchester Centre for Genomic Medicine)

Measuring specific oxysterols (Cholestane-3,5,6-triol) in Wolman disease is a relatively new biomarker for monitoring worsening disease. All oxysterol samples were collected depending on clinical need, and to monitor clinical status. The oxysterol Cholestane-3,5,6-triol (C-triol) was measured in plasma by LCMS/MS using previously published protocols [7, 24]. The activity of LAL (acid esterase) was measured in mixed leukocytes using the artificial substrate 4-methylumbelliferyl-palmitate based in the previously published protocol [25].

### Survival analysis

The small sample size in this study is reflects the rarity of Wolman disease, and therefore no formal sample size calculation was performed. To facilitate comparison, data from other studies has been included in the comparator arms. The historical, untreated arm comprised of 21 patients described by Jones et al. 2017 [15] and their data is included here with permission from the lead author. The ERT arm includes combined data from the recently published VITAL and CL08 studies, including 9 and 10 patients respectively [13]. It should be noted that some patients within the ERT arm received HCT in their treatment course so overlap with the multimodal group is likely. The proportion of surviving patients has been estimated by KaplanMeier analysis using Graph Pad.

## Data Availability

Datasets supporting conclusions of this article are included within the article.

## Abbreviations

ADA: Anti drug antibodies
CRIM: Cross reactive immunological material
DSR: Diet substrate reduction
ERT: Enzyme replacement therapy
HCT: Hematopoietic stem cell transplant
HLH: Hemophagocytic lymphohistiocytosis
HSC-GT: Hematopoietic stem cell gene therapy
LAL: Lysosomal acid lipase
RMCH: Royal Manchester Childrens Hospital
